# Perinatal factors impacting echocardiographic left ventricular measurement in small for gestational age infants: a prospective cohort study

**DOI:** 10.1186/s12887-023-04204-w

**Published:** 2023-08-08

**Authors:** Ibrahim Elmakaty, Ahmed Amarah, Michael Henry, Manoj Chhabra, Danthanh Hoang, Debbie Suk, Nitin Ron, Beata Dygulska, Farrah Sy, Madhu B. Gudavalli, Ali M. Nadroo, Pramod Narula, Ashraf Gad

**Affiliations:** 1 College of Medicine, QU Health, Qatar University, Doha, Qatar; 2 Pediatrix of Maryland, Rockville, MD USA; 3 Division of neonatal-Prenatal medicine, Department of Pediatrics, New York Presbyterian Brooklyn Methodist Hospital, 506 6th St, Brooklyn, New York 11215 USA; 4 Division of neonatal-Prenatal Medicine, Women’s Wellness and Research Centre, NICU, Hamad Medical Corporation, Doha, Qatar

**Keywords:** Small for gestational age, Neonatal, Echocardiography, Left ventricular dimensions, Perinatal, Appropriate for gestational age

## Abstract

**Introduction:**

Infants born small for gestational age (SGA) have an increased risk of developing various cardiovascular complications. While many influencing factors can be adjusted or adapt over time, congenital factors also have a significant role. This study, therefore, seeks to explore the effect of perinatal factors on the left ventricular (LV) parameters in SGA infants, as assessed immediately after birth.

**Methods and materials:**

This single-center prospective cohort study, conducted between 2014 and 2018, involved healthy SGA newborns born > 35 weeks’ gestation, delivered at New York-Presbyterian Brooklyn Methodist Hospital, and a gestational age (GA)-matched control group of appropriate for gestational age (AGA) infants. Data analysis was performed using multivariate linear regression in STATA.

**Results:**

The study enrolled 528 neonates, 114 SGA and 414 AGA. SGA infants exhibited a mean GA of 38.05 weeks (vs. 38.54), higher male representation (69.3% vs. 51.5%), lower birth weight (BW) (2318g vs 3381g), lower Apgar scores at birth, and a higher rate of neonatal intensive care unit admission compared to AGA infants (41.2% vs.18.9%; *p*<0.001). Furthermore, SGA infants were more likely to be born to nulliparous women (63.16% vs. 38.16%; *p*<0.001), with lower body mass index (BMI) (29.8 vs. 31.7; *p*=0.004), a lower prevalence of gestational maternal diabetes (GDM) (14.9 % vs. 35.5%; *p*<0.001), and a higher prevalence of preeclampsia (18.4 % vs. 6.52%; *p*<0.001). BW was identified as the most significant predictor affecting most LV parameters in this study (*p*<0.001), except shortening fraction, asymmetric interventricular septal hypertrophy and Inter-ventricular septal thickness/LV posterior wall ratio (IVS/LVPW). Lower GA (coefficient = -0.09, *p*=0.002), insulin use in GDM (coefficient = 0.39, *p*=0.014), and low APGAR scores at 1 minute (coefficient = -0.07, *p*<0.001) were significant predictors of IVS during diastole (R-squared [R^2^]=0.24). High maternal BMI is marginally associated with LVPW during systole (R^2^=0.27, coefficient = 0.01, *p*=0.050), while male sex was a significant predictor of LV internal dimension during diastole (R^2^=0.29, *p*=0.033).

**Conclusion:**

This study highlights the significant influence of perinatal factors on LV parameters in SGA infants, with BW being the most influential factor. Although LV morphology alone may not predict future cardiovascular risk in the SGA population, further research is needed to develop effective strategies for long-term cardiovascular health management in this population.

## Introduction

Small for gestational age (SGA) infants have been shown to be at an increased risk of perinatal morbidity and mortality. To our interest, several observational studies have found a link between numerous heart pathologies and SGA compared to appropriate for gestational age (AGA) during fetal life, infancy, and adolescence [1–3]. A longitudinal study conducted in Finland found that young adults who were born SGA had higher blood pressure, impaired glucose metabolism, and increased carotid intima-media thickness (IMT) compared to individuals who were born with normal birth weight (BW) [4]. Another Chinese study found that children born prematurely with intrauterine growth restriction (IUGR) have increased systemic arterial stiffness and mean blood pressure [5].

The increased risk of cardiovascular disease (CVD) in SGA individuals is thought to be related to fetal programming, a concept that describes how the developing fetus responds to environmental cues and adapts its physiology to cope with anticipated postnatal conditions [6]. Adverse conditions during fetal development can result in permanent changes in the structure and function of organs, including the cardiovascular system, leading to an increased risk of CVD [7]. Several mechanisms have been proposed to explain the association between SGA and CVD, including impaired angiogenesis and vasculogenesis, altered structure and function of the heart, and changes in the metabolic and endocrine systems [8]. Many cardiac parameters have been studied over the years using echocardiography (echo) to assess heart function and reveal pathological diseases such as cardiomyopathy [9]. The left ventricle (LV) is the engine that powers the human body's systemic circulation, and echo is used to estimate the LV mass (LVmass) and the LVmass to volume ratio (LVmass/vol) to predict pathologies like LV hypertrophy [10]. A prospective, population-based, longitudinal cohort study found that being born prematurely or with a very low BW is associated with differences in cardiovascular structure and function in adulthood when assessed at the ages of 26-30, including smaller LV, LV end-diastolic volume, LV end-systolic volume, stroke volume, and cardiac output [11].

Echo is a key tool used to assess these structural and functional changes in the heart, particularly parameters of the LV [12]. Recognizing that LV morphology, as assessed by echo, can undergo modifications due to various factors throughout life, the specific alterations in LV morphology in SGA infants and the precise perinatal factors contribuSting to these changes are not yet fully understood. This knowledge gap necessitates further investigation to provide more understanding of the cardiac health trajectory in SGA infants.

This study aims to investigate the relationship between perinatal factors and echocardiographic LV parameters in SGA infants, measured postnatally, to provide a more comprehensive understanding of the of the potential factors that might be associated LV parameters.

## Materials and methods

### Study design and setting

This investigation was a single-center, prospective cohort study aimed at identifying factors influencing echocardiographic estimates of LV function in SGA infants compared to AGA infants. The study was carried out at the labor ward and neonatal intensive care unit (NICU) of NewYork-Presbyterian Brooklyn Methodist Hospital (NYPBMH) between 2014 and 2018. As part of this study, comprehensive echo evaluations were conducted on all newborns within the time frame of 48 to 72 hours following delivery and before hospital discharge. This specific period was chosen to assess the cardiac function and characteristics of the infants. The study was conducted in adherence to the Strengthening the Reporting of Observational Studies in Epidemiology guidelines [13] and the Helsinki declaration. Ethical approval was obtained from the hospital's institutional review board. Data confidentiality was ensured by assigning unique identification numbers to each participant and securing data in a locked repository.

### Eligibility criteria

SGA newborns who appeared healthy and were delivered at the NYPBMH were included in this study. SGA status was determined using Fenton growth charts [14]. SGA was defined as a birth weight below the 10^th^ percentile of gestational age using Fenton growth charts [14]. All SGA infants underwent echo examination per unit protocol to assess for congenital heart disease. The SGA cohort was matched with a control group of AGA infants born during the same period, who had undergone echocardiography before hospital discharge for murmur evaluation. Infants with signifcant cardiac pathologies were excluded, though those with asymptomatic and insignificant benign pathologies (e.g., hemodynamically insignificant or restrictive patent ductus arteriosus, patent foramen ovale) were included. Neonates were excluded if echo was not performed for any reason, if data was lost during follow-up, if they were large for gestational age (LGA), or if they had congenital malformations, perinatal depression, low 5-minute APGAR score (<5), genetic diagnosis, heart disease, hypoxic respiratory failure, severe sepsis/shock requiring vasopressors or inotropes, or if they were born before the 35^th^ week of gestation.

### Study variables

The independent variables in this study, believed to influence LV cardiac function, were classified as categorical or continuous variables. Categorical variables included NICU status, sex, gravidity, parity, gestational diabetes mellitus (GDM), preeclampsia, ethnicity, mode of delivery. Continuous independent variables encompassed gestational age (GA), BW, height, head circumference (HC), Ponderal Index (PI) = (Weight in grams) / (Length in centimeters)^3^, maternal age, maternal body mass index (BMI), and APGAR scores at one and five minutes. GDM was diagnosed based on the diagnostic criteria established by the American Diabetes Association, which oral glucose tolerance test with specific plasma glucose thresholds for fasting, 1-hour, and 3-hour measurements [15]. Relevant maternal medical history was obtained from the hospital's electronic medical records, while neonatal information was collected after birth. The target LV cardiac parameters in this study included LVmass, LVmass/vol, inter-ventricular septal thickness during diastole (IVSd) and systole (IVSs), LV internal dimension during diastole (LVIDd) and systole (LVIDs), LV posterior wall thickness at end of diastole (LVPWd) and systole (LVPWs), IVSd/LPVWd ratio, shortening fraction (FS). The only categorical variable among these was asymmetric interventricular septal hypertrophy (ASH), defined as an IVS/LVPW ratio > 1.3. Two-dimensional (2D) Echo evaluation was performed using a Philips 5500 ECHO machine, with a focus on LV dimensions. LV morphology was assessed using the 2D method for structural evaluation and the M-mode method for functional assessment. Echocardiography in this study was performed by a single board-certified cardiologist.

### Data analysis

Data were organized using Microsoft Excel and presented in tables. Raw data were presented as frequencies and percentages or means and Standard Error (SE) as appropriate. Differences in baseline characteristics between categorical variables were analyzed using the chi-square test of independence, and the two-sample t-test was used for continuous variables. Statistical significance was set at *p*<0.05.

To explore the relationship between the continuous LV cardiac variables and the independent variables of interest, a linear regression model was employed. This model allows for the examination of the nature and strength of the relationship between these variables. Initially, each independent variable was fitted individually (univariate analysis) to evaluate potential significance. Subsequently, significant variables were combined in a multivariate linear regression model, eliminating collinear variables based on correlation matrix (>0.9), variance inflation factors (>10), or clinical relevance. For the multivariate linear regression analysis, we included all enrolled infants, encompassing both the SGA and AGA groups, in order to examine the combined effects of various factors on the outcome variable. As part of sensitivity analysis, a repeat of the same multivariate linear regression analysis will be done on SGA and AGA groups separately. The results of the multivariate linear regression were reported, including coefficients, standard errors, *p*-values, R-squared (R^2^) and adjusted (adj) R^2^. Given the binary nature of ASH, binary logistic regression was employed. All statistical analyses, tables, and graphs were generated using Stata software (version 16.0, StataCorp LLC, College Station, TX).

## Results

### Selection and inclusion process

**Fig. 1:**
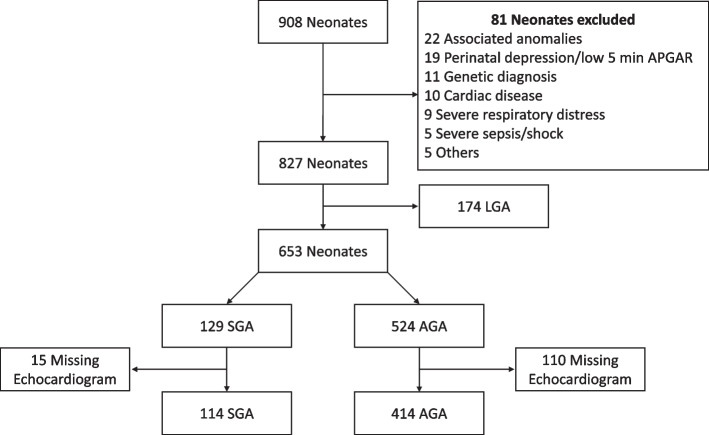
Inclusion process for our study participants (provided at the end as separate PowerPoint file)

Figure 1 depicts the process of selection and inclusion in our prospective cohort study. During the study period, 908 neonates were admitted to the hospital. Because of the pathologies described in Fig. 1, 81 neonates were excluded, and 174 neonates were found to be LGA and were appropriately excluded, leaving 653 neonates eligible for our study. 125 neonates were excluded because their echocardiograms were either not performed or were not reported. Our study ultimately included 528 neonates, including 114 SGA and 414 AGA neonates.

## Baseline characteristics

Table 1 summarizes the differences in baseline characteristics between SGA and AGA in various categorical variables. The table shows that a significantly higher proportion of SGA infants required admission to the NICU compared to AGA infants (41.23% vs. 17.87%; *p*<0.001). Additionally, SGA infants had a higher percentage of males (69.30% vs. 51.45%; *p*=0.001) and a lower prevalence of maternal diabetes (14.91% vs. 35.51%; *p*<0.001) and higher preeclampsia (18.42% vs. 6.52%; *p*<0.001) compared to AGA infants. In terms of parity, SGA infants had a significantly higher proportion of nulliparous mothers (63.16% vs. 38.16%; *p*<0.001) and a lower proportion of multiparous mothers (34.21% vs. 58.21%) compared to AGA infants. There was no significant difference in mode of delivery between the two groups. Regarding maternal ethnicity, the largest proportion of SGA infants was born to White and African American mothers (39.47%), while White mothers accounted for the majority of AGA infants (43.24%). Despite these trends, the difference in ethnicity between the two groups was not statistically significant (*p*=0.070).

**Table 1:** Differences in baseline characteristics between SGA and AGA in categorical variables

**Variable**	**SGA (*n*=114)**		**AGA (*n*=414)**		**Total**	***P*-value**
	**N**	**%**	**N**	**%**		
NICU Admission						
No	50	45.9	237	60.2	287	0.008
Yes	60	54.1	157	39.8	216	
Gender						
Male	79	69.3	213	51.45	292	0.001
Female	35	30.7	201	48.55	236	
Apgar scores						
1- minute Apgar <7	20	17.5	40	9.7	60	0.02
1- minute Apgar ≥7	94	82.5	372	90.3	466	
5- minute Apgar <7	7	6.1	4	1	11	<0.001
5- minute Apgar ≥7	107	93.9	408	99	515	
Gravidity						
Primigravida	45	39.47	93	22.46	138	0.005
Multigravida	51	44.74	242	58.45	293	
Grand multigravida*	15	13.16	73	17.63	88	
Parity						
Nulli-parity	72	63.16	158	38.16	230	<0.001
Multiparity	39	34.21	241	58.21	280	
Grand multiparity*	2	1.75	12	2.9	14	
Gestational Diabetes						
No	96	84.21	259	62.56	355	<0.001
Yes	17	14.91	147	35.51	164	
Preeclampsia						
No	92	80.7	383	92.51	475	<0.001
yes	21	18.42	27	6.52	48	
Ethnicity						
White ethnicity	45	39.47	179	43.24	224	0.07
African American	45	39.47	114	27.54	159	
Hispanic	4	3.51	8	1.93	12	
Asian	3	2.63	15	3.62	18	
Other*	3	2.63	31	7.49	34	
Delivery More						
Vaginal	63	55.26	204	49.28	267	0.249
CS*	50	43.86	207	50	257	

Abbreviations: *SGA* Small for Gestational Age, *AGA* Appropriate for Gestational Age, *NICU* Neonatal Intensive Care Unit, *CS* Cesarean section, *MOD* Mode Of Delivery

^*^Missing data: NICU admission (*n*=25), gravida (*n*=9), parity (*n*=4), diabetes status (*n*=9), preeclampsia status (*n*=5), ethnicity (*n*=81), MOD (*n*=4)

Table 2 shows the differences in baseline characteristics SGA and AGA in several continuous variables. The mean GA for SGA babies was lower than AGA babies (38.05 weeks vs 38.69 weeks, *p*<0.001), however, the difference is clinically insignificant. BW, height, head circumference, and chest circumference were all significantly lower in SGA babies compared to AGA babies (*p*<0.001). PI was also significantly lower in SGA babies (2.44 g/cm^3^ vs 2.79 g/cm^3^, *p*<0.001). Maternal BMI was also significantly higher in AGA babies than SGA babies (29.82 kg/m^2^ vs 31.68 kg/m^2^, *p*=0.004). In terms of neonatal outcomes, SGA babies had a significantly lower APGAR score at 1 minute (7.73 vs 8.29, *p*=0.001) and a significantly lower APGAR score at 5 minutes (8.53 vs 8.83, *p*<0.001). There were no significant differences in maternal systolic and diastolic blood pressure or mean blood pressure between the two groups.

**Table 2:** Differences in baseline characteristics between SGA and AGA in continuous variables

**Variable**	**SGA**			**AGA**			***P*-value**
	**Mean ± SD**	**95 % CI**		**Mean ± SD**	**95 % CI**		
Gestational age (weeks)	38.05 ± 1.90	37.70	38.41	38.69 ± 1.48	38.54	38.83	<0.001
Birth weight (g)	2317.91 ± 446.78	2235.01	2400.82	3381.05 ± 445.77	3337.99	3424.12	<0.001
Birth Height (cm)	45.59 ± 3.05	45.03	46.16	49.5 ± 2.64	49.24	49.75	<0.001
Birth HC (cm)	31.79 ± 1.86	31.44	32.13	34.31 ± 1.91	34.12	34.49	<0.001
Birth CC (cm)	28.55 ± 2.35	27.98	29.11	33.11 ± 2.01	32.88	33.35	<0.001
Ponderal Index (g/cm^3^)	2.44 ± 0.35	2.38	2.51	2.79 ± 0.34	2.76	2.82	<0.001
Maternal age (years)	30.57 ± 5.89	29.47	31.67	31.55 ± 5.77	30.99	32.11	0.111
Maternal BMI (kg/m^2^)	29.82 ± 6.08	28.68	30.95	31.68 ± 6.03	31.09	32.27	0.004
Maternal BP (mmHg)							
Systolic	124.59 ± 15.36	121.37	127.81	123.01 ± 14.35	121.62	124.41	0.352
Diastolic	75.86 ± 12.60	73.22	78.49	74.39 ± 10.84	73.34	75.44	0.26
Mean	80.89 ± 91.08	63.92	97.87	90.2 ± 11.72	89.06	91.34	0.449

Abbreviations: SGA, Small for Gestational Age; AGA, Appropriate for Gestational Age; HC, Head Circumference; CC, Chest Circumference; BMII, body mass index; SD, Standard deviation; CI, Confidence Interval

### LV dependent variables comparison between SGA and AGA infants

Table 3 presents a summary of LV dependent variables in SGA and AGA infants. The results indicate that SGA infants have significantly lower mean values for IVSd (3.47 ± 0.63 mm vs. 4.00 ± 0.78 mm, *p*<0.001), IVSs (4.51 ± 0.81 mm vs. 5.34 ± 1.05 mm, *p*<0.001), LVIDd (16.82 ± 2.06 mm vs. 18.57 ± 2.11 mm, *p*<0.001), LVIDs (10.95 ± 1.47 mm vs. 11.93 ± 1.62 mm, *p*<0.001), LVPWd (3.09 ± 0.61 mm vs. 3.48 ± 0.61 mm, *p*<0.001), LVPWs (4.20 ± 0.59 mm vs. 4.82 ± 0.70 mm, *p*<0.001), LVmass (7.50 ± 2.35 g vs. 10.27 ± 3.26 g, *p*<0.001), and Lvmass/Vol (44.82 ± 11.02 g/m^2^ vs. 49.49 ± 12.15 g/m^2^, *p*<0.001) compared to AGA infants. There was also a significant difference in the mean values for FS between SGA and AGA infants (34.48 ± 4.10% vs. 35.56 ± 4.51%, *p*=0.021).

**Table 3:** Differences in LV parameters between SGA and AGA

**Variable**	**SGA**			**AGA**			***P*-value**
	**Mean ± SD**	**95 % CI**		**Mean ± SD**	**95 % CI**		
IVSd (mm)	3.47 ± 0.63	3.36	3.59	4.00 ± 0.78	3.93	4.08	<0.001
IVSs (mm)	4.51 ± 0.81	4.37	4.67	5.34 ± 1.05	5.24	5.44	<0.001
LVIDd (mm)	16.82 ± 2.06	16.43	17.2	18.57 ± 2.11	18.37	18.78	<0.001
LVIDs (mm)	10.95 ± 1.47	10.67	11.22	11.93 ± 1.62	11.78	12.09	<0.001
LVPWd (mm)	3.09 ± 0.61	2.98	3.21	3.48 ± 0.61	3.42	3.54	<0.001
LVPWs (mm)	4.20 ± 0.59	4.09	4.31	4.82 ± 0.70	4.76	4.9	<0.001
IVS/LVPW ratio	1.14 ± 0.27	1.09	1.18	1.17 ± 0.28	1.14	1.20	0.246
FS (percentage)	34.48 ± 4.10	33.71	35.24	35.56 ± 4.51	35.12	36.00	0.021
LVmass (g)	7.50 ± 2.35	7.06	7.94	10.27 ± 3.26	9.96	10.59	<0.001
LVmass/Vol (g/m^2^)	44.82 ± 11.02	42.76	46.89	49.49 ± 12.15	48.3	50.67	<0.001

Abbreviations: *LVmass* Left Ventricular mass, *LVmass/Vol* LVmass to volume ratio, *IVSd* Inter-Ventricular Septal thickness during diastole, *IVSs* Inter-Ventricular Septal thickness during systole, *LVIDd*, LV Internal Dimension during diastole, *LVIDs* LV Internal Dimension during systole, *LVPWd* LV Posterior Wall thickness at end of diastole, *LVPWs* LV Posterior Wall thickness at end of systole, *IVS/LVPW* Inter-Ventricular Septal thickness to LV Posterior Wall thickness ratio in diastole, *FS* Shortening Fraction, *SD* Standard Deviation

Table 4 shows a subgroup analysis of our SGA population based on growth restriction status. We had 70 symmetric IUGR neonates and 44 asymmetric IUGR neonates. Our analysis reveals no statistically significant differences in the LV cardiac parameters measured. The comparison of demographic findings between the symmetric IUGR and asymmetric IUGR groups revealed no significant differences, except for head circumference (symmetric IUGR 30.9±0.20 cm vs asymmetric IUGR 33.1±0.20 cm, *p*<0.001) and APGAR score at 1 minute (symmetric IUGR 8.1±0.19 vs asymmetric IUGR 7.1±0.38, *p*=0.014). The p-values for NICU (*p*=0.381), sex (*p*=0.143), gravida (*p*=0.232), parity (*p*=0.970), GDM (*p*=0.837), preeclampsia (*p*=0.683), ethnicity (*p*=0.113), mode of delivery (*p*=0.552), insulin (*p*=0.208), GA (*p*=0.403), BW (*p*=0.739), height (*p*=0.080), chest circumference (*p*=0.886), ponderal index (*p*=0.141), maternal age (*p*=0.719), and maternal BMI (*p*=0.325) were non-significant.

**Table 4:** Differences in LV parameters between symmetric and asymmetric IUGR

**Variable**	**Symmetric IUGR**			**Asymmetric IUGR**			***P*-value**
	**Mean ± SD**	**95 % CI**		**Mean ± SD**	**95 % CI**		
IVSd (mm)	3.41 ± 0.60	3.27	3.55	3.57 ± 0.68	3.37	3.78	0.183
IVSs (mm)	4.49 ± 0.84	4.29	4.69	4.57 ± 0.78	4.33	4.80	0.613
LVIDd (mm)	16.89 ± 2.20	16.36	17.41	16.70 ± 1.84	16.15	17.26	0.650
LVIDs (mm)	10.94 ± 1.55	10.57	11.31	10.95 ± 1.35	10.54	11.36	0.978
LVPWd (mm)	3.07 ± 0.65	2.91	3.22	3.14 ± 0.55	2.97	3.30	0.548
LVPWs (mm)	4.18 ± 0.58	4.04	4.32	4.25 ± 0.61	4.07	4.43	0.515
IVS/LVPW ratio	1.12 ± 0.26	1.06	1.19	1.16 ± 0.27	1.07	1.24	0.526
FS (percentage)	34.69 ± 4.34	33.66	35.73	34.14 ± 3.71	33.01	35.26	0.483
LVmass (g)	7.47 ± 2.51	6.87	8.07	7.55 ± 2.08	6.92	8.19	0.852
LVmass/Vol (g/m^2^)	45.17 ± 12.27	42.20	48.14	44.28 ± 8.84	41.59	46.97	0.677

Abbreviations: *IUGR* Intra-uterine Growth Restriction, *LVmass* Left Ventricular mass, *LVmass/Vol* LVmass to Volume ratio, *IVSd* Inter-Ventricular Septal thickness during diastole, *IVSs* Inter-Ventricular Septal thickness during systole, *LVIDd* LV Internal Dimension during diastole, *LVIDs* LV Internal Dimension during systole, *LVPWd* LV Posterior Wall thickness at end of diastole, *LVPWs* LV Posterior Wall thickness at end of systole, *IVS/LVPW* Inter-Ventricular Septal thickness to LV Posterior Wall thickness ratio in diastole, *FS* Shortening Fraction, *SD* Standard Deviation

### Main analysis results

The findings of the study, which used multivariate linear regression analysis, are presented in Table 5. In the IVSd regression model (R^2^=0.24, Adj R^2^=0.23), GA had a negative and significant effect, with a coefficient of -0.09 (*p*=0.002). APGAR score at 1 minute had also statistically significant negative effect on IVSd, with a coefficient of -0.07 (*p*<0.001). Maternal insulin use during pregnancy had a positive and significant effect on IVSd with a coefficient of 0.39 (*p*=0.014). As for BW, it was significantly associated positively with IVSd and IVSs (*p*<0.001).

**Table 5:** Associations of perinatal factors with LV parameters

**LV parameter**	**N**	**Variable**	**Coeff**	**SE**	***P***	**R**^**2**^**, Adj R**^**2**^
**IVSd**	514	GA	-0.09	0.03	0.002	0.24, 0.23
		Birth weight	0.00	0.00	<0.001	
		Category	0.10	0.06	0.073	
		APGAR1	-0.07	0.02	<0.001	
		Insulin use	0.39	0.16	0.014	
*Other variables controlled for in this model: PI, Maternal BMI						
** IVSs**	265	Birth weight	0.00	0.00	<0.001	0.19, 0.17
*Other variables controlled for in this model: NICU admission, GA, Birth weight, Category, PI, Maternal BMI, Diabetes, Diabetic control, Preeclampsia						
** LVIDd**	266	NICU admission	-0.54	0.31	0.082	0.29, 0.27
		Sex	-0.62	0.29	0.033	
		Birth weight	0.00	0.00	<0.001	
*Other variables controlled for in this model: GA, Category, PI, Maternal BMI, APGAR1, Preeclampsia, Mean BP						
** LVIDs**	272	NICU admission	-0.37	0.22	0.092	0.18, 0.16
		Sex	-0.36	0.21	0.098	
		Birth weight	0.00	0.00	0.005	
*Other variables controlled for in this model: GA, Category, PI, Preeclampsia						
** LVPWd**	260	Birth weight	0.00	0.00	<0.001	0.28, 0.26
		PI	-0.19	0.11	0.094	
*Other variables controlled for in this model: NICU admission, GA, Category, Gravidity, Maternal BMI, Preeclampsia, Insulin use						
** LVPWs**	269	Birth weight	0.00	0.00	<0.001	0.27, 0.25
		Maternal BMI	0.01	0.01	0.050	
*Other controlled for variables in this model: NICU admission, GA, Category, PI, Preeclampsia						
** FS**	518	PI	0.06	0.03	0.076	0.027, 0.019
		MOD	0.69	0.40	0.083	
*Other variables controlled for in this model: Birth weight, Category.						
** LVmass**	269	Birth weight	0.00	0.00	<0.001	0.32, 0.30
*Other variables controlled for in this model: NICU admission, Sex, GA, Category, PI, Parity, Maternal BMI, Preeclampsia						
** LVmass/Vol**	508	Birth weight	0.01	0.00	<0.001	0.08, 0.07
*Other variables controlled for in this model: GA, Category, PI, Maternal BMI, Insulin use						

Abbreviations: *LVmass* Left Ventricular mass, LVmass/Vol LVmass to Volume ratio, *IVSd* Inter-Ventricular Septal thickness during diastole, *IVSs* Inter-Ventricular Septal thickness during systole, *LVIDd* LV Internal Dimension during diastole, *LVIDs* LV Internal Dimension during systole, *LVPWd* LV Posterior Wall thickness at end of diastole, *LVPWs* LV Posterior Wall thickness at end of systole, *IVS/LVPW* Inter-Ventricular Septal thickness to LV Posterior Wall thickness ratio in diastole, *FS* Shortening Fraction, *SD* Standard Deviation, *RMSE* Root Mean Square Error, *Coeff* Coefficient, *NICU* Neonatal Intensive Care Unit, *GA* Gestational Age, *PI* Ponderal Index, *BMI* Body Mass Index, *BP* Blood Pressure, *MOD* Mode Of Delivery, *Adj* Adjusted

Table 5 also shows LVIDd regression results (R^2^=0.29, Adj R^2^=0.27). Sex was found to be a significant predictor of LVIDd (*p*=0.033), with a negative coefficient of -0.62, indicating that male infants have a higher LVIDd than female infants. In LVIDs regression model (R^2^=0.18, Adj R^2^=0.16), BW was the only significant variable in it (*p*<0.001). BW was significantly associated with LVIDd (*p*<0.001), LVIDs (*p*=0.005) and LVPWd (*p*<0.001). Maternal BMI was found to be marginally significant (*p*=0.05), with a positive coefficient of 0.01, indicating that higher maternal BMI is associated with an increase in LVPWs (R^2^=0.27, Adj R^2^=0.25). LVPWs was also significantly associated with BW (*p*<0.001).

The regression model for LVmass had an R^2^ of 0.32 and Adj R^2^ of 0.30, and BW was found to be a significant predictor of LVmass (*p*<0.001), with a positive coefficient, indicating that a higher BW is associated with an increase in LVmass. As for LVmass/Vol regression model (R^2^=0.08, Adj R^2^=0.07), no significant relationships were observed between variables assessed and LVmass/Vol other than that of BW (*p*<0.001). No significant relationships were observed between either neonatal or maternal factors and FS. In the univariate binary regression, ASH and IVS/LVPW showed no significant associations with the included independent variables; thus, the results of that analysis are not presented.

Table 6 presents a comprehensive analysis of the associations between perinatal factors and LV parameters, examining SGA and AGA infants separately. The findings reveal significant associations in several instances. Specifically, in the SGA group, a significant association was observed between IVSd and BW (*p*=0.002). Similarly, significant associations were found between LVIDd and the Ponderal Index (*p*=0.038). Conversely, LVIDs exhibited a significant association with Birth Weight (*p*=0.020). Furthermore, Birth Weight demonstrated a significant association with LVPWd (*p*=0.001), while Maternal BMI exhibited a significant association with LVPWs (*p*=0.010). However, no significant associations were identified between FS and any of the examined variables. Additionally, BW demonstrated a significant association with LVmass (*p*<0.001), whereas LVmass/Vol exhibited a significant association with Maternal BMI (*p*=0.045). As for the AGA group, IVSd showed a significant association with GA (*p*=0.002), BW (*p*<0.001), APGAR at 1 minute (*p*<0.001) and maternal insulin use during pregnancy (p=0.035). Similarly, IVSs exhibited a significant association with Birth Weight (*p*=0.005). For LVIDd, significant associations were found with Sex (*p*=0.008) and Birth Weight (*p*=0.007), whereas LVIDs showed significant associations with Sex (*p*=0.040) and BW (*p*=0.039). Furthermore, Birth Weight demonstrated significant associations with LVPWd (*p*<0.001), LVPWs (*p*<0.001), LVmass (*p*<0.001) and LVmass/Vol (*p*=0.001).

**Table 6:** Associations of perinatal factors with LV parameters in SGA and AGA separately

**Small for Gestational Age**							**Appropriate for Gestational Age**						
**LV parameter**	**N**	**Variable**	**Coeff**	**SE**	**P**	**R**^**2**^**, Adj R**^**2**^	**LV parameter**	**N**	**Variable**	**Coeff**	**SE**	**P**	**R**^**2**^**, Adj R**^**2**^
**IVSd**	112	GA	-3.03	0.55	0.528	0.20, 0.16	**IVSd**	402	GA	-0.10	0.32	0.002	0.18, 0.17
		Birth weight	0.00	0.00	0.002				Birth weight	0.00	0.00	<0.001	
		APGAR1	-0.02	0.29	0.591				APGAR1	-0.09	0.24	<0.001	
		Insulin use	0.55	0.61	0.365				Insulin use	0.36	0.17	0.035	
*Other variables controlled for in these models: PI, Maternal BMI													
**IVSs**	57	Birth weight	0.00	0.00	0.150	0.33, 0.24	**IVSs**	208	Birth weight	0.00	0.00	0.005	0.07, 0.03
*Other variables controlled for in these models: NICU admission, GA, Birth weight, PI, Maternal BMI, Diabetes, Diabetic control, Preeclampsia													
**LVIDd**	56	NICU admission	-1.16	0.79	0.148	0.44, 0.33	**LVIDd**	209	NICU admission	-0.44	0.35	0.214	0.20, 0.16
		Sex	0.75	0.60	0.219				Sex	-0.89	0.33	0.008	
		Birth weight	0.00	0.00	0.090				Birth weight	0.00	0.00	0.007	
		PI	-1.72	0.81	0.038				PI	-0.01	0.51	0.971	
*Other variables controlled for in these models: GA, Maternal BMI, APGAR1, Preeclampsia, Mean BP													
**LVIDs**	58	NICU admission	-0.17	0.55	0.755	0.36, 0.29	**LVIDs**	214	NICU admission	-0.37	0.25	0.139	0.11, 0.08
		Sex	0.38	0.44	0.391				Sex	-0.51	0.26	0.040	
		Birth weight	0.00	0.00	0.020				Birth weight	0.00	0.00	0.039	
*Other variables controlled for in these models: GA, PI, Preeclampsia.													
**LVPWd**	55	Birth weight	0.00	0.00	0.001	0.46, 0.38	**LVPWd**	205	Birth weight	0.00	0.00	<0.001	0.17, 0.13
		PI	-0.04	0.20	0.840				PI	-0.25	0.14	0.069	
*Other variables controlled for in these models: NICU admission, GA, Gravidity, Maternal BMI, Preeclampsia, Insulin use													
**LVPWs**	57	Birth weight	0.00	0.00	0.147	0.37, 0.29	**LVPWs**	212	Birth weight	0.00	0.00	<0.001	0.13, 0.10
		Maternal BMI	0.02	0.01	0.010				Maternal BMI	0.01	0.01	0.343	
*Other variables controlled for in these models: NICU admission, GA, PI, Preeclampsia													
**FS**	112	PI	0.41	1.21	0.732	0.017, -0.01	**FS**	408	PI	-0.36	0.68	0.598	0.027, 0.019
		MOD	0.90	0.78	0.254				MOD	0.88	0.45	0.052	
*Other variables controlled for in these models: Birth weight, Category.													
**LVmass**	57	Birth weight	0.00	0.00	<0.001	0.63, 0.56	**LVmass**	269	Birth weight	0.00	0.00	<0.001	0.32, 0.30
*Other variables controlled for in these models: NICU admission, Sex, GA, PI, Parity, Maternal BMI, Preeclampsia													
**LVmass/Vol**	110	Birth weight	0.01	0.00	0.566	0.10, 0.06	**LVmass/Vol**	508	Birth weight	0.01	0.00	0.001	0.08, 0.07
		Maternal BMI	0.35	0.18	0.045				Maternal BMI	0.058	0.10	0.571	
*Other variables controlled for in these models: GA, PI, Maternal BMI, Insulin use													

Abbreviations: *LVmass* Left Ventricular mass, *LVmass/Vol* LVmass to Volume ratio, *IVSd* Inter-Ventricular Septal thickness during diastole, *IVSs* Inter-Ventricular Septal thickness during systole, *LVIDd* LV Internal Dimension during diastole, *LVIDs* LV Internal Dimension during systole, *LVPWd* LV Posterior Wall thickness at end of diastole, *LVPWs* LV Posterior Wall thickness at end of systole, *IVS/LVPW* Inter-Ventricular Septal thickness to LV Posterior Wall thickness ratio in diastole, *FS* Shortening Fraction, *SD* Standard Deviation, *RMSE* Root Mean Square Error, *Coeff* Coefficient, *NICU* Neonatal Intensive Care Unit, *GA* Gestational Age, *PI* Ponderal Index, *BMI* Body Mass Index, *BP* Blood Pressure, *MOD* Mode Of Delivery, *Adj* Adjusted

## Discussion

In this prospective cohort study, a total of 528 neonates were enrolled, including 114 classified as SGA. The study findings indicate that SGA infants exhibit smaller LV dimensions and mass compared to their AGA counterparts. However, despite the statistically significant differences, both groups demonstrated similar FS values within the normal range, suggesting that the systolic function of the LV remains unaffected by the reduced LV size and mass in SGA infants. The mean LVmass/Vol ratio was 48.48 (SGA=44.82 ± 11.02 g/m^2^ vs. AGA=49.49 ± 12.15 g/m^2^), with no significant associations observed between the assessed variables and LVmass/Vol, except for BW. GA and maternal insulin use were identified as significant predictors of IVSd, while BW and APGAR scores were significant predictors of IVSs. Overall, this study provides valuable insights into the differences in neonatal outcomes and cardiac characteristics between SGA and AGA infants.

Our finding indicted that BW serves as the most reliable predictor of LV dimension, as it was significantly associated with LVmass, LVmass/Vol, LVIDd, LVIDs, IVSd, IVSs, LVPWd, and LVPWs, all demonstrating a positive coefficient. This suggests that a higher BW corresponds with an increase in these parameters. These association could potentially indicate that the size of the LV chamber is directly proportional to the weight of infants, a relationship previously observed in literature [16]. A prior single-center cross-sectional study on 20 SGA children at 24 months revealed that SGA babies had early and mild cardiovascular dysfunction compared to AGA controls, with these changes closely associated with BW [17]. Additionally, that same group have demonstrated that breastfeeding significantly benefits the cardiovascular system [17]. Moreover, in a study of 62 asymmetric IUGR newborns, 39 symmetric IUGR neonates, and a control group of 50 AGA, it was observed that, aside from LVPW in diastole, all LV dimensions were smaller in asymmetric IUGR newborns compared to symmetric IUGR neonates [18]. A similar study used 2D Echo to measure the valve diameters of 376 infants born < 32 weeks gestation and weighing < 2,000 g to give reference values for cardiac valve annulus diameters [19]. They demonstrated a modest relationship between BW and valve diameter, with good intraobserver and interobserver agreement [19]. Another research group tracked SGA newborns for three months using echocardiography on postnatal day five, as well as at one and three months, and found reduced ventricular diameters, ventricular wall thicknesses, and LVmass, but no differences in systolic and diastolic functioning [20].

Our hypothesis posits that the alterations observed are not merely confined to the neonate period but potentially extend into later stages of life. In a study involving a cohort of 64 extremely low BW children aged 11, there were notable differences in LV end-diastolic dimension, LV end-systolic dimension, aorta dimension, and left atrial (LA) dimension , when compared to a control group of 36 healthy children [2]. Another study examining 81 children born as extremely low BW (ELBW) infants with a median BW of 890 g found no patients with diastolic or systolic problems, but there were statistical differences in right ventricle dimension in diastole, LV inner dimension in diastole, and the LA [21]. Additionally, ELBW children exhibited significantly elevated heart rates and higher nocturnal blood pressure levels [21].

The impact of GA on the IVSd reveals a noteworthy finding. GA demonstrated a significant negative effect on IVSd, as indicated by a coefficient of -0.09. Unlike BW, GA displayed an inverse relationship with IVS thickness. This suggests that neonates born prematurely may not necessarily exhibit the manifestations of low birth weight or small size, as prematurity itself is an indication of their condition. Furthermore, premature delivery before the completion of nephrogenesis and IUGR contribute to the development of chronic kidney disease and subsequently lead to CVD [22]. In the case of SGA fetuses and fetuses with IUGR, the myocardial performance index was significantly elevated compared to appropriately grown fetuses, implying impaired cardiac function [23]. Significant distinctions between term and preterm neonates were observed in various cardiac parameters, such as interventricular septum and left systolic/diastolic ventricle diameters, LVPWD in systole (p<0.01), FS and ejection fraction, and basal LV and right ventricular lateral wall measurements in the Ew [24].

Furthermore, APGAR at 1 minute was significantly associated with IVSd (p<0.001), with a negative coefficient indicating that a lower APGAR at 1 minute is associated with increased thickness of IVSd. A low APGAR score is a known risk factor for poor prenatal outcome, which may be related to cardiac effects. The utilization of the 1-minute Apgar score in this study was based on its significance in assessing the initial condition of newborns and its ability to reflect the effects of intrauterine stress factors during birth, whereas the 5-minute Apgar score primarily reflects the efficacy of resuscitative measures performed during the first few minutes of life. This observation is supported by one study showing that the incidence of perinatal complications is higher in infants with Apgar scores less than 7 [25]. This association is explained by the possibility that a low APGAR score indicates fetal distress, premature birth, meconium-stained amniotic fluid, placental abruption, fetal edema, maternal use of certain medications during pregnancy, such as beta-blockers, and certain medical conditions, such as maternal hypotension, anemia, or infections [26–29]. Our analysis also revealed a significant difference (*p*=0.014) in the APGAR score at 1 minute between symmetric and asymmetric IUGR groups. This finding suggests that asymmetric IUGR neonates experienced significantly more stress during their time in the uterus compared to symmetric IUGR neonates.

The sex of the infant was also found to be a significant predictor of LVIDd (*p*=0.033), with a negative coefficient of -0.62, indicating that male infants have a higher LVIDd than female infants. Males appear to have larger LV dimensions than females. According to one research paper, men had significantly higher LV mass, volume, and dimension compared to women, even after adjusting for body size differences [30]. Another study found that men had larger LV end-diastolic and end-systolic volumes compared to women [10].

Maternal insulin consumption during pregnancy has been shown in our study to have a substantial effect on IVSd (R^2^=0.24), with a coefficient of 0.39 (*p*=0.014). Cardiovascular abnormalities are among the most prevalent in diabetes mothers' babies, accounting for 3%-9% of diabetic pregnancies and being 2.5-10 times more common than in normal pregnancies [31]. If the mother has gestational diabetes and develops insulin resistance in the third trimester, the relative risk for serious cardiovascular problems is highest [32]. The incidence of complications was observed to be 3.4% with maternal HbA1c levels less than 8.5% and 22.4% with HbA1c levels greater than 8.5% [33]. Infants born to mothers who had HbA1c levels above 10% in late pregnancy are more likely to suffer neonatal problems [33]. Hypertrophic cardiomyopathy (HCM) is thought to arise as a result of both prenatal hyperinsulinemia and the typically elevated expression and affinity of insulin receptors, resulting in the proliferation and hypertrophy of cardiac myocytes [34, 35]. A case report of cardiac hypertrophy in an exceptionally low BW newborn who received insulin therapy after developing chronic hyperglycemia due to parenteral nourishment supports the concept that iatrogenic hyperinsulinemia plays a role in the development of HCM [36]. Fetal hyperinsulinemia and insulin-like growth factor-I (IGF-1) have also been linked to morphological fetal heart abnormalities. IGF-1 stimulates cardiomyocyte hypertrophy, resulting in reduced myocardial compliance and functional impairment [37, 38]. Nonetheless, the usage of insulin may be an indication of poor glycemic control, which may be a potential confounder in this scenario that leads to IVSd enlargement [39].

We also found that maternal BMI was marginally significant in our study (*p*=0.050), with a positive coefficient of 0.01, indicating that higher maternal BMI might be associated with an increase in LVPWs. Maternal obesity has been linked to changes in the structure and function of the heart, resulting in cardiac abnormalities [40]. The current obesity pandemic among women of childbearing age increases the risk of cardiovascular disease and cardiomyopathies [41]. Pregnancy induces metabolic changes, which are more pronounced in obese women, such as increased body weight, circulation lipids, glucose, and inflammatory markers. Epidemiological studies show that maternal obesity increases the risk of cardiovascular disease and premature mortality in adult and elderly children [42]. Little is known about cardiac development and function in children born to obese mothers, although research indicates that neonates' LV mass increases in proportion to maternal gestational weight growth [43].

Overall, these findings elucidate the connections between perinatal factors such as BW, GA and maternal health conditions, and their influence on specific measures of LV structure and function. This study enhances our comprehension of the baseline cardiac characteristics seen in SGA neonates, taking into account these perinatal influences.

There are several limitations to this study that must be considered when interpreting the findings. Several factors may have an impact on the generalizability of this study. To begin, the study was conducted in a single hospital. Because the study population was unique to this hospital, the results may not be representative of other populations [44]. The study's small sample size may have limited statistical power, resulting in the failure to detect significant differences between groups. The small sample size may have also introduced bias into the study, affecting its external validity [45]. The generalizability is also influenced by exclusion criteria, which may limit the findings' applicability to neonates with specific pathologies. Furthermore, the study's ability to reflect the long-term outcomes of SGA infants' LV function and the overall cardiovascular risk is limited by the lack of long-term follow-up of the participants after their discharge from the hospital. The study was also not blinded, which could lead to bias in measurements and interpretation of the findings [46]. We also didn't include information about potential confounding variables like maternal smoking, which is a known risk factor for low BW and cardiac dysfunction [47]. Finally, echo was used exclusively to evaluate LV function. While echo is a common tool for assessing LV function, it is not without limitations [48]. It is also important to note that our study did not employ advanced imaging techniques for assessing LV morphology. A potential limitation of this study is that the echocardiographic evaluations were conducted by a single cardiologist, which may introduce limitations in assessing interobserver variability.

## Conclusion

This prospective cohort study compared the neonatal maternal data and multiple LV dimension parameters of SGA and AGA infants. All LV dimension parameters were found to be significantly related to BW, implying that a higher BW is associated with an increase in LV dimensions. We also discovered that low APGAR scores at 1 minute were linked to higher IVSd, implying that low APGAR scores may be linked to increased cardiac thickness. Furthermore, we discovered that IVSd is significantly thickened in neonates born to moms who took insulin to control their diabetes during pregnancy, and that male infants had a greater LVIDd than female infants. The changes seen during the neonatal period may have long-term consequences. As a result, it is critical to closely monitor and manage neonates with low BW and low APGAR scores to avoid long-term complications. Further research is needed to explore the long-term implications of these findings and develop appropriate interventions to minimize the risk of adverse cardiac outcomes in neonates.

## Data Availability

The data used for the analysis in this work are available upon reasonable request from the corresponding author.

## Abbreviations

Adj R^2^: Adjusted R-squared
AGA: Appropriate for gestational age
aRR: Adjusted relative risk
ASH: Asymmetric interventricular septal hypertrophy
BMI: Body mass index
BW: Birth weight
CI: Confidence intervals
CVD: cardiovascular disease
ELBW: Extremely low birth weight
FS: Fractional shortening
GA: Gestational age
HC: Head circumference
HCM: Hypertrophic cardiomyopathy
IGF-1: Insulin-like growth factor-I
IMT: Intima-media thickness
IUGR: Intrauterine growth restriction
IVSd: Inter-ventricular septal thickness during diastole
IVSs: Inter-ventricular septal thickness during systole
LGA: Large for gestational age
LV: Left ventricle
LVIDd: LV internal dimension during diastole
LVIDs: LV internal dimension during systole
LVmass: Left ventricular mass
LVmass/vol: Left ventricular mass to volume ratio
LVPWd: LV posterior wall thickness at end of diastole
LVPWs: LV posterior wall thickness at end of systole
NICU: Neonatal intensive care unit
PI: Ponderal Index
R^2^: R-squared
RMSE: Root mean square error
SE: Standard error
SGA: Small for gestational age
